# Prokaryotic Communities Vary with Cultivation Modes of Shrimp (*Litopenaeus vannamei*)

**DOI:** 10.3390/microorganisms13040881

**Published:** 2025-04-11

**Authors:** Guizhen Li, Guangshan Wei, Jianyang Li, Zongze Shao

**Affiliations:** 1 Key Laboratory of Marine Genetic Resources, Third Institute of Oceanography, Ministry of Natural Resources, Xiamen 361005, China; liguizhen.ok@163.com (G.L.);; 2 State Key Laboratory Breeding Base of Marine Genetic Resources, Xiamen 361005, China; 3 Fujian Key Laboratory of Marine Genetic Resources, Xiamen 361005, China

**Keywords:** *Litopenaeus vannamei*, size fraction, cultivation modes, biofloc technology (BFT), environmental parameters

## Abstract

In response to the growing market demand for *Litopenaeus vannamei*, a variety of single-species, high-density, intensive, and high-yield aquaculture modes have arisen. These aquacultural systems are teeming with microorganisms, which play roles in water quality and host health. To uncover the prokaryotic community composition across cultivation modes, we investigated the prokaryotic community composition at two fractionated sizes in the water of three culture modes of *Litopenaeus vannamei*, including high-level pond culture, biofloc technology (BFT), and pond culture. The 16S rRNA gene high-throughput sequencing results indicated that the taxa particularly enriched by high-level pond culture modes were mainly Deltaproteobacteria, while Alpha- and Gammaproteobacteria and Flavobacteria were enriched in the BFT culture modes. The pond culture enriched Bacteroidetes, Sphingobacteriia, Actinobacteria, and Cyanobacteria. PCoA analysis showed that for the pond samples, there were significant differences in the community composition compared with the samples from the other two modes. However, the high-level pond and biofloc samples showed similar community compositions. Furthermore, Canonical Correspondence Analysis (CCA) and Variance Partitioning Analysis (VPA) revealed that NH_4_^+^-N, salinity (Sal), and NO_3_^−^-N were key factors affecting the aquaculture communities.

## 1. Introduction

As ocean fishery stocks decline on a global scale, the aquaculture industry has witnessed exponential growth in recent decades [1,2]. Shrimp farming, being an important component of aquaculture, has experienced rapid development on a global scale in recent years. Since 1987, China has become the world leader in shrimp farming [3]. With the growing competition in the aquaculture industry and people’s increasing attention to food safety, identifying how to cultivate safe, healthy, and green shrimp has become a major challenge for the aquaculture industry [4,5,6].

Whiteleg shrimp, scientifically known as *Litopenaeus vannamei* and also referred to as white shrimp, is a species of the *Penaeus* genus that is native to the eastern coast of the Pacific Ocean. Due to its rapid growth rate, high adaptability, and strong vitality, it has become one of the most productive shrimp species in global aquaculture.

In the aquaculture environment of *Litopenaeus vannamei*, microorganisms are present in substantial quantities. These microorganisms are pivotal in facilitating the transformation and decomposition of harmful substances within the aquaculture system [7,8,9]. Researchers conducted in-depth research on the microbial community structure related to nitrogen and sulfur cycles in shrimp culture water. They found that in traditional and improved culture systems, the number of ammonia-oxidizing bacteria increased with the increase in culture days and remained unchanged in the sediments of low-density culture ponds [10,11,12]. Moreover, researchers conducted a detailed comparative analysis of the microbial community composition in different fractionated sizes of biofloc culture water and found that the fractionated size of biofloc was closely related to the microbial community composition [13,14]. Therefore, analyzing the structure and microbial communities in the aquaculture water of *Litopenaeus vannamei* is of great significance for promoting healthy aquaculture, improving the aquaculture environment, and enhancing the quality of aquaculture water.

Currently, studies examining the composition of aquatic communities in different aquaculture modes are relatively scarce, and the theoretical basis for biological regulation in the culture process is rather weak. Therefore, in this study, we conducted an investigation of culture water and the prokaryotic community at two particle levels across three culture modes. High-level pond farming typically involves artificially constructed ponds, such as those made with plastic lining or concrete, with higher stocking densities, more meticulous management, and, consequently, higher yields. In contrast, pond culture involves natural ponds or artificially excavated earth ponds, with relatively lower stocking densities, more extensive management, less human intervention, lower costs, and lower yields. Biofloc technology is an innovative aquaculture method that uses microbial communities to improve the water quality in aquaculture systems and provides an additional nutritional source for farmed organisms. This method is particularly suitable for high-density farming systems, effectively managing the organic waste produced during the farming process, reducing environmental pollution, and enhancing aquaculture efficiency. The investigation was conducted through high-throughput sequencing (Miseq) of the V4 variable region of the 16S rRNA gene. This study will enhance our understanding of the diversity of prokaryotic communities across the three shrimp culture waters, the diversity of prokaryotic communities at two particle levels, the similarities and differences in prokaryotic communities in the three culture modes, and the key environmental factors that cause the differences in prokaryotic communities among the three culture modes. Thus, it will provide a theoretical basis from a microbial perspective for the biological control of culture water and the selection of culture modes.

## 2. Materials and Methods

### 2.1. Water Sampling

Fujian is a major fishery province, where shrimp farming began as early as the 1970s. Zhangzhou City ranks high in the province in terms of shrimp aquaculture production and area. Therefore, this study selected three mature and stable shrimp farms in Zhangpu and Zhao’an counties, Zhangzhou City, Fujian Province, to collect seawater samples. The culture modes of the three shrimp aquafarms were high-level pond culture (abbreviation, H), biofloc technology culture (abbreviation, B), and pond culture (abbreviation, P), and all of them were in the late stage of culture. For each type of shrimp farm, three shrimp ponds were randomly selected for sample collection, and the seawater extracted from each shrimp farm before entering the ponds was selected as control samples. Excluding the control samples, three ponds were randomly selected as biological replicates for each aquafarm, and water samples were collected from each pond beneath the water surface (0–0.5 m). Each water sample was collected using a sterile ladle. A total of 12 water samples (1 sample/pond × 3 ponds/aquafarm × 3 aquafarms + 3 control samples), with approximately 5 L of water per sample, were collected in aseptic bottles. All the samples were stored in an ice box and immediately transferred to the laboratory. All the samples were filtered within 24 h [13]. The filtration volume applied to each filter membrane was 100 mL. A single filter membrane for each sample was utilized specifically for the extraction of metagenomic DNA. Detailed information on each sample is shown in Table S1 and Figure S1.

### 2.2. Size-Fractionated Filtration and Physiochemical Determination

To determine the relationships between microbial communities and the size of bioflocs, a size-fractionated strategy was employed. Briefly, the water samples were successively filtered through two different membranes with pore sizes of 3 μm and 0.22 μm (Millipore, Burlington, MA, USA) on a clean bench. To minimize filter clogging or particle dislodging issues, the filtration process was carried out at very low speed and pressure, and the filters were changed frequently [15]. As a result, each sample was separated into two size fractions based on the suspended biofloc sizes, covering ranges of 0.22–3 μm (free-living) and >3 μm (particle-attached). The filtered membranes were stored at −20 °C until DNA extraction was performed.

Within 24 h of collection, the chemical parameters, including ammonium, nitrate, nitrite, total nitrogen concentrations, and chemical oxygen demand (COD), were measured in the laboratory according to the standard approaches outlined in Table S2.

### 2.3. DNA Extraction

Total genomic DNA was extracted from three modes of seawater samples using the commercial PowerWater DNA Isolation Kit (MoBio, Carlsbad, CA, USA), following the manufacturer’s instructions meticulously. The quality and quantity of the extracted genomic DNA were assessed using 1% (*w*/*v*) agarose gel electrophoresis and a Nanodrop 2000 Spectrophotometer (Thermo Scientific, Waltham, MA, USA), respectively.

### 2.4. High-Throughput Sequencing of Microbial 16S rRNA Genes

To comprehensively detect both bacterial and archaeal communities, we employed an improved 16S rRNA gene universal primer pair, specifically, the 515F modified and 806R modified primers [16], for high-throughput sequencing. The details of these primers and the PCR conditions are listed in Table S3, while the comprehensive procedures for library construction and high-throughput sequencing are provided in the Supplementary File. The raw sequencing data were deposited in the National Omics Data Encyclopedia database under the accession number SUB00040735.

### 2.5. Data Processing and Statistical Analyses

The raw fastq data were demultiplexed and quality-filtered using QIIME 2 [17], and the detailed parameters are provided in the Supplementary File. Subsequently, at a 97% sequence similarity cutoff, Operational Taxonomic Units (OTUs) were clustered using UPARSE (version 7.0.1090) [18], and chimeric sequences were checked and removed by UCHIME [19]. The representative sequences were classified by the RDP Classifier [20] against the SILVA 132 database [21] using a confidence threshold of 0.7. The OTUs with only one sequence (singleton) and classified as Mitochondria, Chloroplast, or unclassified domain were removed from the data. Finally, the number of sequences was rarefied according to the sample with the least sequence number (1,541,083) for further analyses.

Alpha diversity indexes (Chao1 and Shannon) were calculated from the rarefied OTU table using mothur (http://www.mothur.org) [22]. Bray–Curtis and UniFrac-based β-diversity were calculated using the R ‘vegan’ package (https://vegandevs.github.io/vegan/, accessed on 26 February 2025) and QIIME 2, and plots of principal coordinate analysis (PCoA) were drawn by the ‘ggplot2’ package in R (https://r-charts.com/ggplot2/, accessed on 26 February 2025). Adonis, the Mantel test, and cluster analyses were carried out through the ‘vegan’ package. Boxplots were drawn using the ‘ggplot2’ package, and related one-way analysis of variance (ANOVA) was performed with SPSS Statistic software. The linear discriminant analysis (LDA) effect size (LEfSe) method was used to identify the biomarkers in each group [23]. The phylogenetic tree of core and biofloc-related OTUs was constructed using MEGA software 7.0 [24] and further modified in an online tool (iTOL) [25]. Prokaryotic metagenomic inferences were reconstructed using PICRUSt 2 [26] based on the KEGG Orthology (KO) (https://www.genome.jp/kegg/, accessed on 26 February 2025) and the Clusters of Orthologous Groups of proteins (COGs) databases (https://www.ncbi.nlm.nih.gov/research/cog/, accessed on 26 February 2025) with the rarefied OTU table as inputs. Multivariate regression tree (MRT) analysis was performed using the ‘mvpart’ package in R (version 1.6-2), and a process of 1000 cross-validations was performed to decrease the structure complexity of the tree to predict the main relationship between the microbial data and the variables. Network analyses were carried out using the molecular ecological network analyses (MENA) pipeline at the OTU level for each group with different size fractions [27]. According to the “Best practices for co-occurrence network construction and inference” [28], the Jaccard similarity values were first calculated between each sample of the group using the ‘vegan’ package, and the samples that had lower Jaccard similarity with most of the other samples were excluded from further analyses. The low present OTUs that existed in less than half of the samples of each group were also removed to increase the statistical power of the networks. After that, using the default settings and the recommended similarity threshold of MENA, networks were constructed and visualized in Cytoscape version 3.7.1 [29]. The keystone taxa were defined according to a previous description, which included the connectors, module hubs, and network hubs [27].

## 3. Results

### 3.1. Water Parameters of the Aquafarms

Environmental parameters, including temperature, salinity, pH, dissolved oxygen (DO), ammonium concentrations, nitrate concentrations, nitrite concentrations, total nitrogen (TN), and chemical oxygen demand (COD) were detected in time (Table S2). The results indicated that the inorganic nitrogen levels were generally exceeded in all three aquafarms, with the highest ammonia nitrogen levels in the high-level pond aquafarm (2.5 mg L^−1^) and the highest nitrate and nitrite concentrations (1.3 mgL^−1^ and 0.77 mg L^−1^) in the biofloc technology aquafarm.

In all three aquafarms, the total nitrogen content was more than 20 mg L^−1^. The highest total nitrogen content was found in the high-level pond aquafarm, with an average of 44.6 mg L^−1^. Except for some inorganic nitrogen (ammonium, nitrate, and nitrite), the main component of the total nitrogen was organic nitrogen, which was mainly protein-based, with most of it originating from the feed inputs and a small portion of it originating from the dead alga, zooplankton, and farmed animals. In addition, the COD_Cr_ content of the samples was 200–300 mg L^−1^ (Table S2), indicating that the three types of aquaculture water had a high organic matter content.

Significant differences between the three aquafarms were tested using one-way ANOVA (Table S2). The outcomes indicated that the temperatures of the high-level pond and biofloc technology aquafarms deviated significantly (*p* < 0.05) from those of the pond aquafarm. Furthermore, the salinity, the nitrate, and the COD concentrations in the pond aquafarm stood out as markedly distinct from both the high-level pond aquafarm and biofloc technology aquafarm. Interestingly, the pH values in the biofloc technology aquafarm were significantly different from both the high-level pond aquafarm and the pond aquafarm. Additionally, the ammonium concentrations in the high-level pond aquafarm were significantly varied compared to both the biofloc technology aquafarm and the pond aquafarm. Moreover, any two of the three aquafarms exhibited significant differences in both the nitrite concentrations and total nitrogen levels. However, it is noteworthy that the dissolved oxygen (DO) levels did not differ significantly (*p* > 0.05) among the three aquafarms. In conclusion, despite some similarities, the environmental parameters of the three aquafarms exhibit substantial differences, which could be an important reason for the differences in culture yields.

### 3.2. α-Diversity Index of Microbial Communities and Distribution of Taxonomic Units

**Figure 1:**
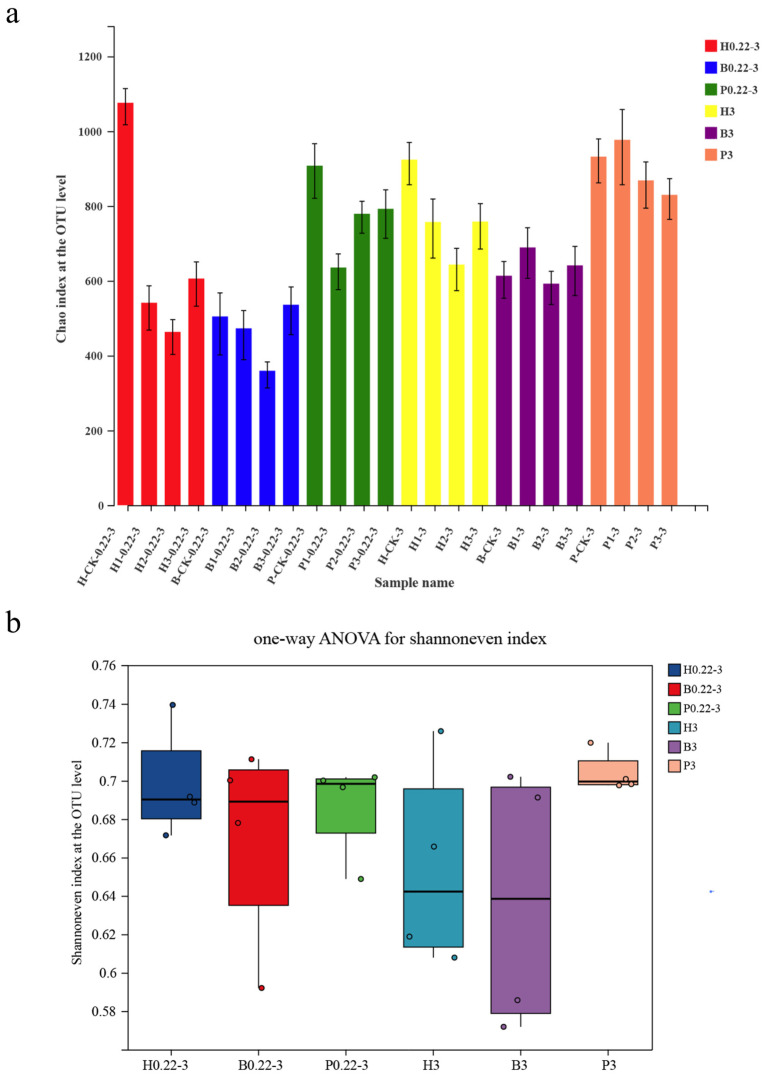
(**a**) Alpha-diversity Chao index at the OTU level of different seawater samples from different aquaculture models. (**b**) One-way analysis of variance (ANOVA) for the Shannon even index followed by Duncan’s multiple range test.

To gain insights into the *α*-diversity of different cultivation models, Chao and Shannon index analyses were conducted at the OTU level. The Chao and Shannon index plots at the OTU level showed a consistent trend between the two methods (Figure 1). For the samples with particle sizes ranging from 0.22 to 3 μm, the highest index was observed in the pond aquafarm samples (P), while the lowest index was found in the biofloc technology aquafarm (B) (Figure 1). This indicated that the pond aquafarm samples exhibited the highest *α*-diversity, whereas the biofloc technology aquafarm samples exhibited the lowest *α*-diversity. Similarly, for samples with particle sizes greater than 3 μm, the highest indexes were also observed in the pond aquafarm samples (as indicated by both Chao and Shannon indexes), and the lowest indexes were found in the biofloc technology aquafarm samples (B) (Figure 1). This further suggests that the pond aquafarm samples have the highest *α*-diversity, while the biofloc technology aquafarm samples have the lowest *α*-diversity for this particle size.

Furthermore, the distribution across different categorical units for each sample was counted (Table S4). A total of 18 bacterial phyla and 41 bacterial orders with clear taxonomic status were detected at both the 0.22–3 μm particle and >3 μm particle levels in the high-level pond samples. Comparing the two particle size samples, the bacterial species diversity was higher in the >3 μm samples. In addition, the archaeal community was detected with only one OTU in the 0.22–3 particles, and the presence of archaea was not detected in the >3 μm particles. In contrast, 198 OTUs were detected in the 0.22–3 μm particles, and 55 OTUs were detected in the >3 μm particles in the control group of the high-level pond samples. It can be seen that the archaeal community was eliminated during the cultural process in the high-level pond. In addition, the bacterial communities, after the high-level pond culture, also became less diverse.

Within the biofloc technology aquafarm samples, 16 bacterial phyla and 36 bacterial orders with clear taxonomic status and 418 OTUs were detected in the 0.22–3 μm particle samples, while the >3 μm particle samples contained 20 bacterial phyla and 39 bacterial orders with clear taxonomic status and an increased count of 540 OTUs.

Furthermore, only two OTUs of archaea were detected in the 0.22–3 μm particles, and one OTU of archaea was detected in the >3 μm particles, whereas four OTUs were detected in the 0.22–3 μm particles, and no archaea were detected in the >3 μm particles in the control of bioflocs samples. This shows that the archaeal community has a very low abundance in shrimp aquaculture waters with biofloc technology. Guangshan Wei [13] also analyzed the community in shrimp aquaculture water with biofloc technology, and the results revealed that the content of archaea was found to be notably low, which is consistent with our results. It is noteworthy that the application of biofloc technology did not lead to a decrease in the diversity of prokaryotic organisms in the cultivated waters; instead, it resulted in a significant increase in this diversity.

The pond culture sample was also examined, revealing 22 bacterial phyla, 50 classes, and 682 OTUs in the 0.22–3 μm particles. Furthermore, the analysis identified 23 bacterial phyla, 52 classes, and 727 OTUs in the >3 μm particles. The diversity of the bacterial community was higher in the >3 μm particles when comparing the two types of samples. Furthermore, the analysis revealed that seven OTUs were identified within the 0.22–3 μm particles, and eight OTUs were present in the >3 μm particles. In contrast, the control group exhibited five OTUs within the 0.22–3 μm particles and six OTUs in the >3 μm particles, indicating a notably diminished archaeal community abundance in both the cultured water of the pond culture and the control water samples.

In conclusion, for all three culture modes, the diversity of bacterial communities was higher in the >3 μm particles than in the 0.22–3 μm particles, which is in agreement with the results reported in other studies [13,30]. It is worth mentioning that the prokaryotic community diversity of both the 0.22–3 μm particles and the >3 μm particles became lower in both the high-level pond culture and the pond culture waters after shrimp aquaculture compared to the control. The biofloc technology mode, on the other hand, was different from the above two modes, and after shrimp aquaculture, the diversity of both the 0.22–3 μm particles and the >3 μm particles became higher compared with the control group. This is the difference between the biofloc technology culture and the other two culture methods. In addition, the archaeal content was minimal, except for the notably higher concentration observed in the control group of the high-level pond. In the study conducted by Guangshan Wei [13], the absence of amoA, a key gene associated with nitrification, was not detected in shrimp aquaculture water, accompanied by a near absence of AOA (ammonia-oxidizing archaea) and AOB (ammonia-oxidizing bacteria) taxa. Therefore, it is reasonable to postulate that archaea may not be the primary contributors to nitrification processes within the shrimp aquaculture ecosystem.

### 3.3. Microbial Community Compositions

**Figure 2:**
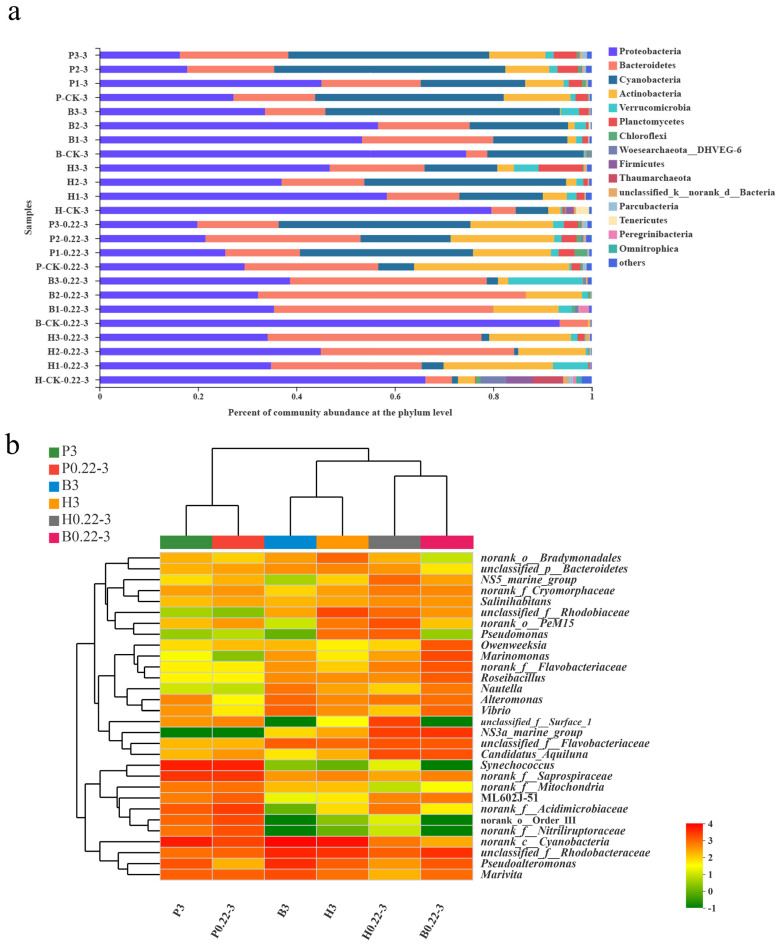
(**a**) Phylum-level dominant bacteria distribution of different seawater samples from the three aquaculture modes. (**b**) Genus-level heatmap analysis of different seawater samples of the different aquaculture modes.

We defined a dominant class as one in which the relative proportion of sequences within any specific group surpasses 1% of the total. Through analysis, there were six dominant phyla in the community composition of the high-level pond samples, with the highest content of Proteobacteria (34.16–58.41%), followed by Bacteroidetes (14.71–43.46%), Cyanobacteria (0.86–41.02%), Actinobacteria (2.05–22.22%), Verrucomicrobia (0.49–7.21%), and Planctomycetes (0.041–9.04%). Seven dominant phyla were present in the biofloc technology sample, namely, Proteobacteria (32.19–56.58%), Bacteroidetes (12.29–54.40%), Cyanobacteria (0–47.63%), Actinobacteria (0.22–13.30%), Verrucomicrobia (1.18–15.22%), Planctomycetes (0.0089–1.95%), and Peregrinibacteria (0–2.02%). Seven dominant phyla existed in the pond culture water sample, namely, Proteobacteria (16.34–45.10%), Bacteroidetes (15.14–31.49%), Cyanobacteria (18.32–46.94%), Actinobacteria, (7.78–21.07%), Verrucomicrobia (1.04–2.13%), Planctomycetes (2.76–4.72%), and Chloroflexi (0.41–2.50%) (Figure 2a). The dominant phylum levels of the three culture modes were largely the same, primarily consisting of Proteobacteria, Bacteroidetes, Cyanobacteria, Actinobacteria, Verrucomicrobia, and Planctomycetes. Other related studies have also found Proteobacteria to be the most dominant bacterial group [13,31,32].

In terms of grain size, Proteobacteria emerged as the dominant phylum in both groups. However, the abundance of Proteobacteria was significantly higher in the granule-attached group. Furthermore, Cyanobacteria were predominantly present in the attached particle group, whereas Bacteroidetes and Actinobacteria were mainly found in the free-living group.

At the genus level, the samples from the groups were averaged to identify the top 30 genera in terms of total abundance. Among these, the genera with clear taxonomic status included *Marivita*, *Pseudoalteromonas*, ML602J-51, *Synechococcus*, *Candidatus_Aquiluna*, NS3a_marine_group, *Vibrio*, *Alteromonas*, *Nautella*, *Roseibacillus*, *Marinomonas*, *Owenweeksia*, *Pseudomonas*, *Salinihabitans*, and NS5_marine_group. The remaining fifteen genera belonged to taxa of uncertain taxonomic status. In addition, AOA (ammonia-oxidizing archaea) were only detected in the control samples of the high-level pond for *Candidatus Nitrosopumilus* but were not detected in any other samples. AOB (ammonia-oxidizing bacteria) were also detected mainly in the control group of the high-level pond with the genera *Nitrosomonas* and *Nitrosospira*. In conclusion, AOA and AOB were present in negligible amounts in all samples except the control group of the high-level pond. In previous studies, it was also mentioned that key genes associated with autotrophic nitrification (amoA) were not detected in a biofloc technology sample and that AOA and AOB taxa were almost absent [13]. Therefore, since AOA and AOB were not dominant in the community, this suggests that autotrophic nitrification was not dominant in the aquaculture water.

Through sample clustering analysis, the two grain size samples (P0.22-3 and P3) from the pond culture converged into a single cluster (Figure 2b). This indicates that the community composition of the pond culture samples was significantly different from that of the other two modes samples. In contrast, the free-living and particle-attached grain samples from the high-level pond and biofloc technology culture samples formed distinct clusters, signifying a similarity in their respective community compositions. Specifically, the free-living grain samples from the high-level pond were similar to that of the free-living grain samples from the biofloc technology, while the particle-attached grain samples from the high-level pond demonstrated a comparable community composition to the particle-attached grain samples from the biofloc technology. This indicates that there are similarities in community composition between the high-level pond technique and the biofloc technology technique.

**Figure 3:**
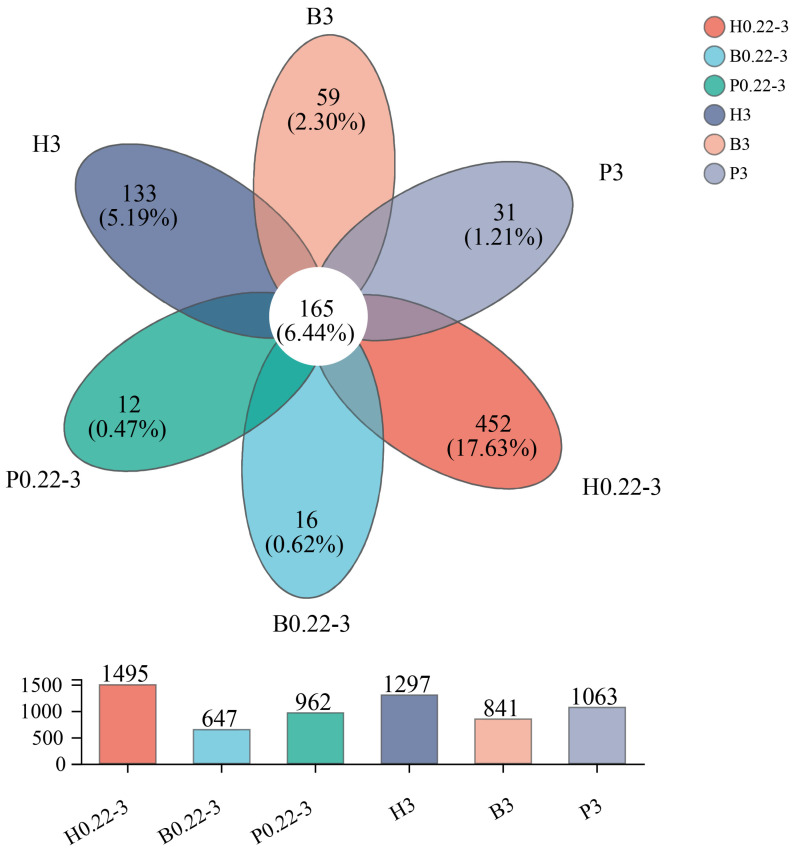
Venn analysis at the OTU level of different seawater samples from the three aquaculture modes.

At the OTU level, the biofloc technology samples had the lowest OTU diversity at both the free-living and particle-attached grain levels. The distribution of unique OTUs for the six groups of samples was 452 (H0.22-3), 16 (B0.22-3), 12 (P0.22-3), 133 (H3), 59 (B3), and 31 (P3). The highest number of unique OTUs (452 vs. 133) was found in the high-level pond samples for both the free-living and particle-attached samples (see Figure 3). This may be related to the relatively open culture pattern in the pond culture.

### 3.4. Differences in Microbial Diversity Between Cultivation Modes

**Figure 4:**
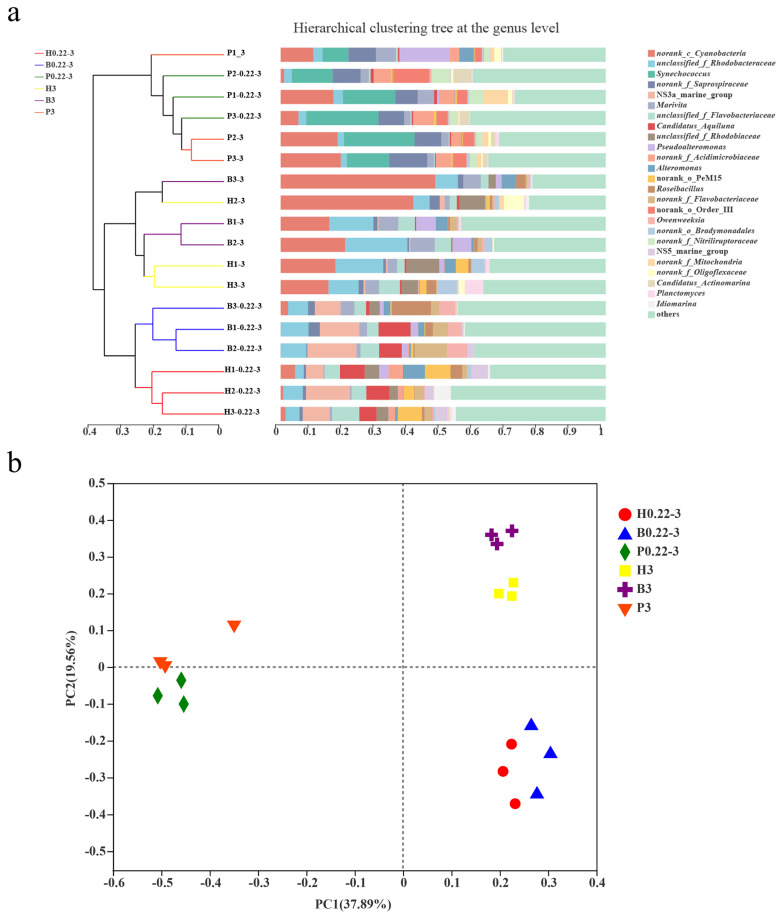
(**a**) Hierarchical clustering analysis at the genus level of different aquaculture seawater samples. (**b**) Principal coordinates analysis at the OTU level of aquaculture seawater samples from the three different aquaculture modes.

To investigate the similarities and differences in community composition among the different samples, a Beta-diversity analysis was conducted to compare the diversity of the microbial communities among the different culturing modes and different grain sizes. Hierarchical clustering analyses at the genus level revealed that parallel samples of free-living and particle-attached grains from the pond culture clustered in a large branch. Distinctly, the parallel samples of free-living grains from the high-level pond and those from the biofloc technology aquafarms were each clustered separately in a small branch, which subsequently merged into a larger branch. Interestingly, the particle-attached grain samples from both the high-level pond and the biofloc technology aquafarms were cross-clustered in one large branch (Figure 4a).

By PCoA analysis, all the pond culture samples were distinctly clustered in a single branch, whereas the free-living grains (0.22–3 μm) from both the high-level pond and biofloc technology aquafarms were clustered together. Additionally, the particle-attached grains (>3 μm) from both the high-level pond and biofloc technology aquafarms were individually segregated into distinct clusters (Figure 4b).

The outcomes of the hierarchical cluster analysis and principal coordinates analysis (PCoA) showed that the community composition of the pond culture samples was significantly different from that of the high-level pond and biofloc technology aquafarm samples, extending to both free-living (0.22–3 μm) and particle-attached (>3 μm) size fractions. However, there existed a degree of similarity in the community composition between the free-living (0.22–3 μm) fractions of the high-level pond samples and the corresponding fractions of the biofloc technology samples, as well as in the particle-attached (>3 μm) fractions of both sample types. Therefore, from the perspective of community composition, the pond culture mode was distinguished from both the high-level pond and biofloc technology culture modes. In contrast, the community composition was similar in both the high-level pond and biofloc technology cultures, regardless of the free-living particle fractions (0.22–3 m) or particle-attached fractions (>3 μm), as long as the particle size was the same. In conclusion, the perspective of the community composition of the high-level pond culture water and the biofloc technology culture water is more similar. This suggests that the pond culture mode is more different from the high-level pond and biofloc technology modes, while the high-level pond and biofloc technology modes are similar in terms of culture and management.

### 3.5. Relationships Between the Microbial Community and Environmental Factors

**Figure 5:**
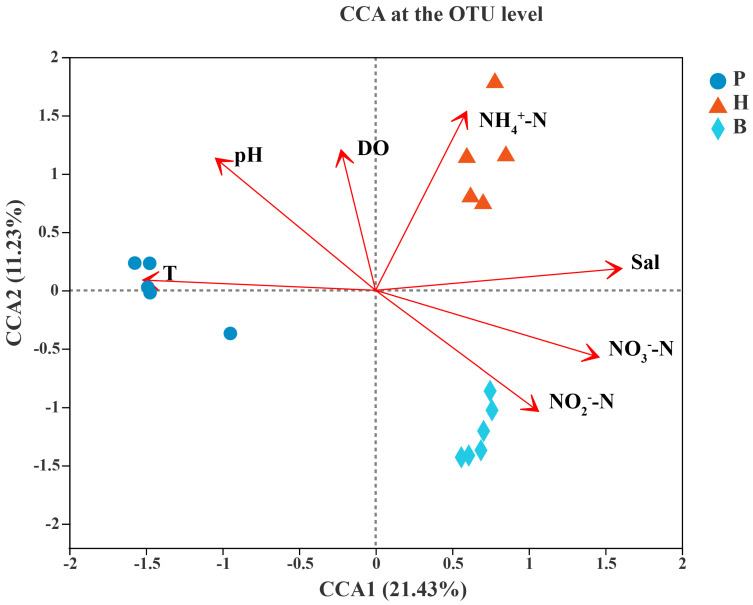
CCA analysis of seawater samples of the three aquaculture modes of *Litopenaeus vannamei*.

To analyze the relationship between environmental factors, samples, and flora, a Canonical Correspondence Analysis (CCA) was conducted. To investigate the effects of external environmental factors on community structure, temperature (T), pH, salinity, dissolved oxygen (DO), ammonia nitrogen (NH_4_^+^-N), nitrate (NO_3_^−^-N), and nitrite (NO_2_^−^-N) were measured at each sampling site. Among the seven environmental parameters, T, pH, Sal, NH_4_^+^-N, NO_3_^−^-N, and NO_2_^−^-N had a significant effect (*p* < 0.001) on the community structure, and the order of the effect of each factor on the community was as follows: Sal > NH_4_^+^-N > NO_3_^−^-N > pH > T > NO_2_^−^-N (Figure 5).

**Figure 6:**
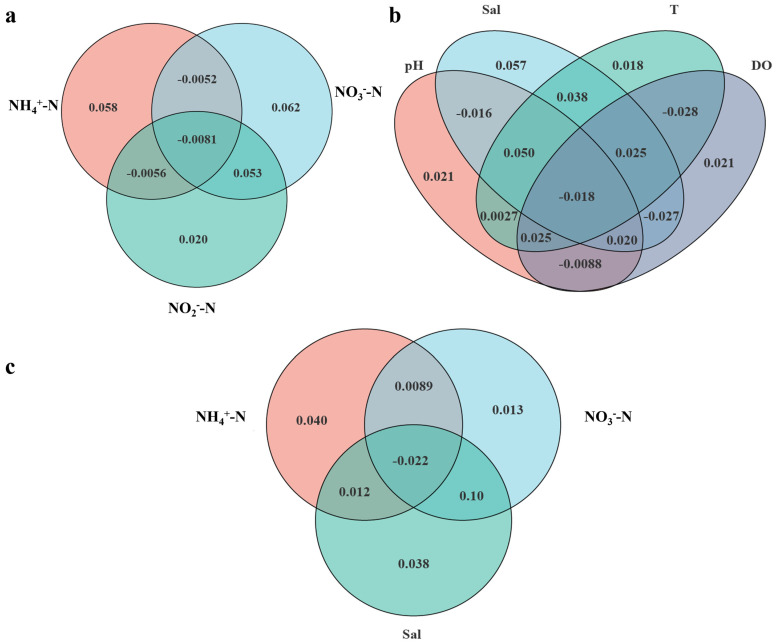
VPA analysis of seawater samples of the three aquaculture modes of *Litopenaeus vannamei.* (**a**) The inorganic nitrogen parameters (NO_3_^−^-N, NH_4_^+^-N, NO_2_^−^-N). (**b**) The environmental parameters (Sal, T, DO, pH). (**c**) The single impacts of environmental factors (Sal, NO_3_^−^-N, NH_4_^+^-N).

In order to further quantitatively assess the degree of explanation of microbial communities by each environmental factor, so as to identify the important environmental factors affecting the differences in the microbial communities, VPA (variation partition analysis) analysis was conducted. For the convenience of analysis, the environmental factors were divided into two groups of inorganic nitrogen (NH_4_^+^-N, NO_3_^−^-N, and NO_2_^−^-N) and environmental parameters (pH, Sal, T, and DO), which were analyzed separately. The results showed that in the inorganic nitrogen group, the environmental factor with the highest degree of explanation for these two environmental factors was NO_3_^−^-N (0.062), followed by NH_4_^+^-N (0.058) and then NO_2_^−^-N (0.020). This indicates that among the inorganic nitrogen parameters, NO_3_^−^-N and NH_4_^+^-N had the greatest influence on the microbial community variations among the single environmental factors, with 6.2% and 5.8%, respectively, and NO_2_^−^-N had a minimal impact, with only 2.0% (Figure 6a). Among the environmental parameters analyzed, salinity (Sal) emerged as the most significant factor, accounting for the highest single explanation of 5.7%, closely followed by pH and dissolved oxygen (DO), each contributing 2.1%, and, finally, temperature (T), with an explanation of 1.8%. That is, when considering the single impacts of environmental factors on community structure, salinity exerted the most pronounced influence (Figure 6b).

In order to further analyze the influence degree of the three most important environmental parameters (NO_3_^−^-N, NH_4_^+^-N, and Sal) on the community structure individually, NO_3_^−^-N, NH_4_^+^-N, and Sal were set up as a group of environmental factors, and a VPA was carried out. The results showed that the three most important environmental parameters individually affected the community in the order of NH_4_^+^-N (4.0%) > Sal (3.8%) > NO_3_^−^-N (1.3%) (Figure 6c). The co-resolution was Sal (13.1%) > NO_3_^−^-N (10.2%) > NH_4_^+^-N (3.9%) (Figure 6c).

In summary, out of the seven environmental factors examined, six pivotal ones—Sal, NH_4_^+^-N, NO_3_^−^-N, pH, T, and NO_2_^−^-N—elicited variations in the bacterial communities across the three different modes of cultured seawater. Notably, NH_4_^+^-N, Sal, and NO_3_^−^-N emerged as the three most significant factors. In terms of single-factor impacts, NH_4_^+^-N exerted the strongest influence, closely followed by Sal and then NO_3_^−^-N. When considering their combined effects, NH_4_^+^-N again led the way, with Sal and NO_3_^−^-N following behind. Regarding their combined influence, salinity (Sal) stood out as the primary driver, with NO_3_^−^-N and NH_4_^+^-N occupying subsequent positions.

**Figure 7:**
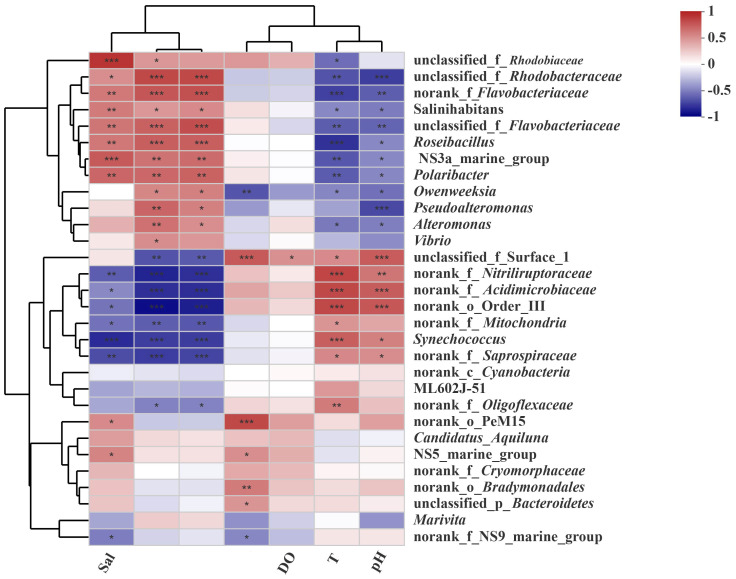
Correlation heatmap analysis of seawater samples of the three aquaculture modes of *Litopenaeus vannamei.* The number of asterisks (*) typically indicates the level of statistical significance. Single * (*p* ≤ 0.05), significant; Double ** (*p* ≤ 0.01), highly significant; Triple *** (*p* ≤ 0.001) extremely significant.

To visualize the relationship between various species and environmental parameters and to assess the correlation between microbial taxa and these parameters, correlation heatmap plots were constructed at the genus level. Among the seven environmental variables, T and pH were consistently correlated with microorganisms, while the NH_4_^+^-N and dissolved oxygen (DO) levels also demonstrated consistent correlations with microorganisms. Furthermore, the NO_3_^−^-N and NO_2_^−^-N concentrations were consistently correlated with the microorganisms (Figure 7).

Among the top 30 genus-level microorganisms, *Salinihabitans*, *Roseibacillus*, NS3a_marine_group, *Polaribacter*_4, *Owenweeksia*, *Pseudoalteromonas*, *Alteromonas*, and *Vibrio* displayed a negative correlation with the environmental factors of temperature (T) and pH, while they were positively correlated with salinity (Sal), NO_3_^−^-N, and NO_2_^−^-N. Conversely, they showed a lesser correlation with NH_4_^+^-N and DO. In contrast, *Synechococcus* was positively correlated with T and pH but negatively with Sal, NO_3_^−^-N, and NO_2_^−^-N. Additionally, *Marivita* was negatively related to Sal, NH_4_^+^-N, and pH, was positively linked to NO_3_^−^-N and NO_2_^−^-N, and demonstrated a weak correlation with T. Researchers uncovered that particles ranging from 10 to 100 μm were significantly correlated with environmental factors, and in biofloc technology culture water, microbial abundance was inversely correlated with T and Sal, while it was positively correlated with NH_4_^+^-N and DO [13].

### 3.6. Network Properties and the Potential Keystone Taxa

**Figure 8:**
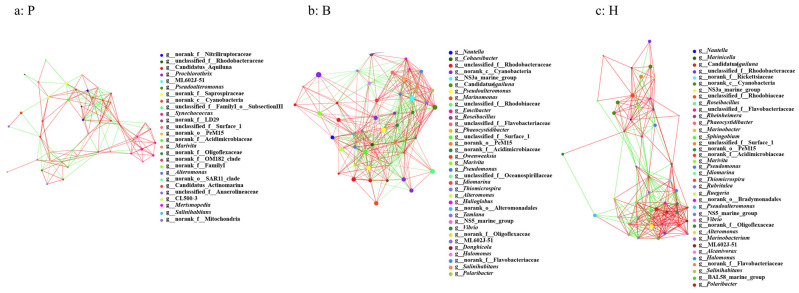
Network analysis of seawater samples of the three aquaculture methods of *Litopenaeus vannamei*.

The analysis of the samples can identify both positive and negative correlations, as well as significant associations, among the interactions between the various species present. Network analyses enable the identification of species coexistence within environmental samples, the interaction among species in the same environment, and the composition of pivotal species, thereby elucidating the underlying mechanisms of the formation of differences between samples [33,34]. Figure 8 illustrates an intricate network diagram of the microbial communities, wherein the line colors symbolize correlation types: red represents positive correlations, while green denotes negative correlations. Each node represents a distinct OTU. Notably, the analysis reveals that the correlations among the species within the communities of the three cultivated water samples are predominantly positive in nature.

The network analysis of the pond samples showed that more than 70% of the species in the top 50 abundances corresponded to OTU clustering coefficients greater than 0.3, and more than 28% of the species corresponded to OTU clustering coefficients greater than 0.6, suggesting that the species in the flocculent samples were not tightly connected with each other. At the genus level, the clustering coefficients of OTUs affiliated with CL500-3 and ML602J-51 surpassed 0.8, suggesting robust interconnectivity and heightened interactions among these OTUs with other species within the community. Consequently, they serve as crucial connecting nodes within the network. Conversely, OTUs corresponding to taxa like norank_c_Cyanobacteria, norank_f_Oligoflexaceae, *Pseudoalteromonas*, norank_f_Saprospiraceae, and unclassified_f_Anaerolineaceae displayed clustering coefficients nearing zero, indicative of minimal interactions with other taxa, thereby diminishing their significance within the community network (Figure 8a).

The network analysis of the floc samples revealed that at the genus level, over 96% of the top 50 abundant species exhibited OTU clustering coefficients exceeding 0.3, with more than 73% displaying coefficients greater than 0.6. This robust interconnectivity suggests that species within the floc samples were tightly bound to each other (Figure 8b). Specifically, OTUs belonging to *Cohaesibacter*, *Salinihabitans*, ML602J-51, *Emcibacter*, unclassified_f_*Oceanospirillaceae*, Candidatus_*Aquiluna*, and *Alteromonas* were found to have cluster coefficients surpassing 0.8. This signifies that these OTUs are not only intimately linked with other species in the community but also engage in robust interactions among themselves, thereby playing a pivotal role in facilitating connections within the microbial network (Figure 8b).

The network analysis of the high-level pond samples revealed that at the genus level, over 90% of the top 50 abundant species presented OTU clustering coefficients exceeding 0.3, and more than 60% boasted coefficients greater than 0.6. This finding underscores the intimate connectivity among species within the high-level pond samples (Figure 8c). Notably, specific species such as norank_c_Cyanobacteria, *Pseudomonas*, and norank_f__Acidimicrobiaceae possessed clustering coefficients above 0.8, indicating their profound interconnectedness within the community network and pivotal roles in facilitating strong interactions among various species. Conversely, OTUs affiliated with unclassified_f__Rhodobacteraceae, *Alteromonas*, and _norank_f__Oligoflexaceae displayed clustering coefficients approaching zero, signifying minimal interaction with other taxa in the community network, thereby contributing to a relatively low level of overall community interactivity (Figure 8c).

Based on the network diagram of species interactions (Figure 8) and the results of the foregoing analyses, it can be concluded that among the species of the three aquaculture microbial communities, the strongest interactions are observed in the biofloc technology samples, followed by the high-level pond samples and then the pond samples. This might be related to the content of particulate matter in the water, which is highest in the biofloc technology culture, followed by the high-level pond culture and then the pond culture. It is hypothesized that the particulate matter in the water provides attachment space for microorganisms and brings species closer together, thereby enhancing interactions. Researchers delved into the microbial communities within the culture water of biofloc technology and discovered that the interactions between species were strengthened with an increase in particle size.

## 4. Discussion

### 4.1. Aquaculture Mode and Particle Size Are Closely Related to the Microbial Community

Numerous studies have concentrated on examining the prokaryotic microbial communities associated with distinct particle sizes across a variety of aquatic environments [13,15,35,36,37]. Within these studies, aquatic prokaryotes are typically divided into two size fractions: free-living (FL, <3 μm) and particle-attached (PA, >3 μm). However, investigations into different particle sizes under different aquaculture modes are relatively scarce. Our work has encompassed a comprehensive examination of three different aquaculture modes and delved into the microbial community structure across both free-living (FL, <3 μm) and particle-attached (PA, >3 μm) particle sizes. Our research has demonstrated that aquaculture mode and particle size are the two predominant factors influencing the abundance, diversity, community structure, functionality, and co-occurrence networks of prokaryotic communities. Given the more extensive management approach of pond culture in contrast to high-level pond and biofloc technology-based high-density aquaculture systems, and with less human intervention, the pond culture microbial community structure is distinct from the other two modes across all particle sizes (Figure 5 and Figure 6). Furthermore, since both high-level ponds and biofloc technology-based culture fall into the category of high-density intensive aquaculture systems, the community structure will be similar when the particle size is the same. In addition, although the abundance of archaea is low, FL tends to be reported in some other aquatic ecosystems [13,38,39,40].

### 4.2. Ammonium, Salinity, and Nitrate Are Three Significant Environmental Factors Influencing the Microbial Community

Microbial communities, which are crucial for ecosystem function and sustainability, are under environmental pressure [41]. Therefore, microbial communities are sensitive to environmental change [32,41,42,43]. The outcomes from the CCA and VPA analyses indicated that of the seven environmental factors considered, salinity, ammonium, nitrate, pH, temperature, and nitrite are the six principal factors at play. Notably, ammonium, salinity, and nitrate are identified as the three key environmental factors that predominantly contribute to the variations observed among the three distinct aquaculture communities (Figure 7 and Figure 8). Ammonium and nitrate are important nitrogen sources for the microbial community [44]. Therefore, they can affect the microbial community structure, which is quite understandable. However, even under marine aquaculture conditions, it is indeed unexpected that salinity still has a significant impact on the microbial community. Studies have proven that salinity is indeed a principal environmental factor influencing the structure of the microbial community [8,13,45]. Some studies suggest that salinity plays a more significant role than geographic distance in shaping microbial diversity and community structure [45]. Our results robustly support this notion. The possible reason is that some key taxa may exhibit more sensitivity to salinity; thus, alterations in salinity can induce changes in the community structure.

## 5. Conclusions

In this research, we explored the diversity of seawater and microbial communities across two fractionated sizes within three distinct shrimp farming systems, i.e., high-level pond culture, biofloc technology culture, and traditional pond culture. Our analysis delved into the community structure composition across different samples, assessing the species diversity within these communities under contrasting cultivation methods and particle sizes. Furthermore, we conducted a network analysis to understand the co-occurrence patterns of community structures and identified the key environmental factors responsible for the observed differences in the community composition across the different farming practices. This comprehensive study aimed to enhance our understanding of the microbial diversity in the three shrimp farming environments, highlighting the diversity at different particle levels and elucidating the similarities and differences in community between the samples from the different culture systems. It also sought to uncover the primary environmental factors that lead to these differences, thus providing a valuable understanding of the complex ecological interactions in these aquaculture settings.

The dominant genera with a distinct taxonomic position and ranking within the top ten for the different samples of the three culture modes are *Marivita*, *Pseudoalteromonas*, *Synechococcus*, *Vibrio*, *Alteromonas*, *Nautella*, *Roseibacillus*, *Marinomonas*, *Owenweeksia*, and *Pseudomonas*. The taxa particularly enriched by high-level pond culture modes are mainly Deltaproteobacteria. The taxa specifically enriched in biofloc technology culture communities are Alphaproteobacteria, Gammaproteobacteria, and Flavobacteria. Meanwhile, the taxa particularly enriched in pond culture modes are Bacteroidetes_Incertae_Sedis, Sphingobacteriia, Actinobacteria, and Cyanobacteria.

The results of the PCoA analysis show that for the pond samples, whether in the free-living fraction or the particle-attached fraction, there are significant differences in the community composition compared with the samples from the other two modes. However, if the particles of the high-level pond samples and the biofloc samples are the same, their community species compositions are similar.

The results of the CCA and VPA analyses show that among the seven environmental factors, there are three key environmental factors that cause differences in the three different aquaculture communities, namely, NH_4_^+^-N, salinity (Sal), and NO_3_^−^-N. Among the single environmental factors’ separate influences on the bacterial community, NH_4_^+^-N has the greatest influence, followed by salinity (Sal) and then NO_3_^−^-N. In terms of the combined influence, salinity (Sal) has the greatest degree, followed by NO_3_^−^-N and then NH_4_^+^-N.

Future investigations could center around probing into the functional aspects of the preponderant genera, namely, *Marivita*, *Pseudoalteromonas*, and *Vibrio*, within the shrimp farming ecological systems. Gaining an in-depth comprehension of how these genera interface with shrimp development, disease resistance capabilities, and the modulation of water quality would offer pivotal understandings for refining and enhancing aquaculture methodologies. To illustrate, experimental setups could be devised to deliberately adjust the population densities of these genera and subsequently monitor the resultant impacts on the health and productivity of shrimp.

## Data Availability

The data presented in this study are available in National Omics Data Encyclopedia database under the accession number SUB00040735.

## Supplementary Materials

The following supporting information can be downloaded at: https://www.mdpi.com/article/10.3390/microorganisms13040881/s1, Figure S1: Station locations of three aquafarms of *Litopenaeus vannamei*. Table S1: Sample source information in this study. Table S2: Physiochemical parameters of the water samples. Table S3: Primers and PCR conditions in this study. Table S4: Number of identified classification levels (phylum to OTU level) in aquaculture seawater samples.
