# Supplementary figures and images for: Prokaryotic Communities Vary with Cultivation Modes of Shrimp (Litopenaeus vannamei)
Source: Microorganisms. 2025 Apr 11;13(4):881. doi: 10.3390/microorganisms13040881 (PMC12029335; doi:10.3390/microorganisms13040881)

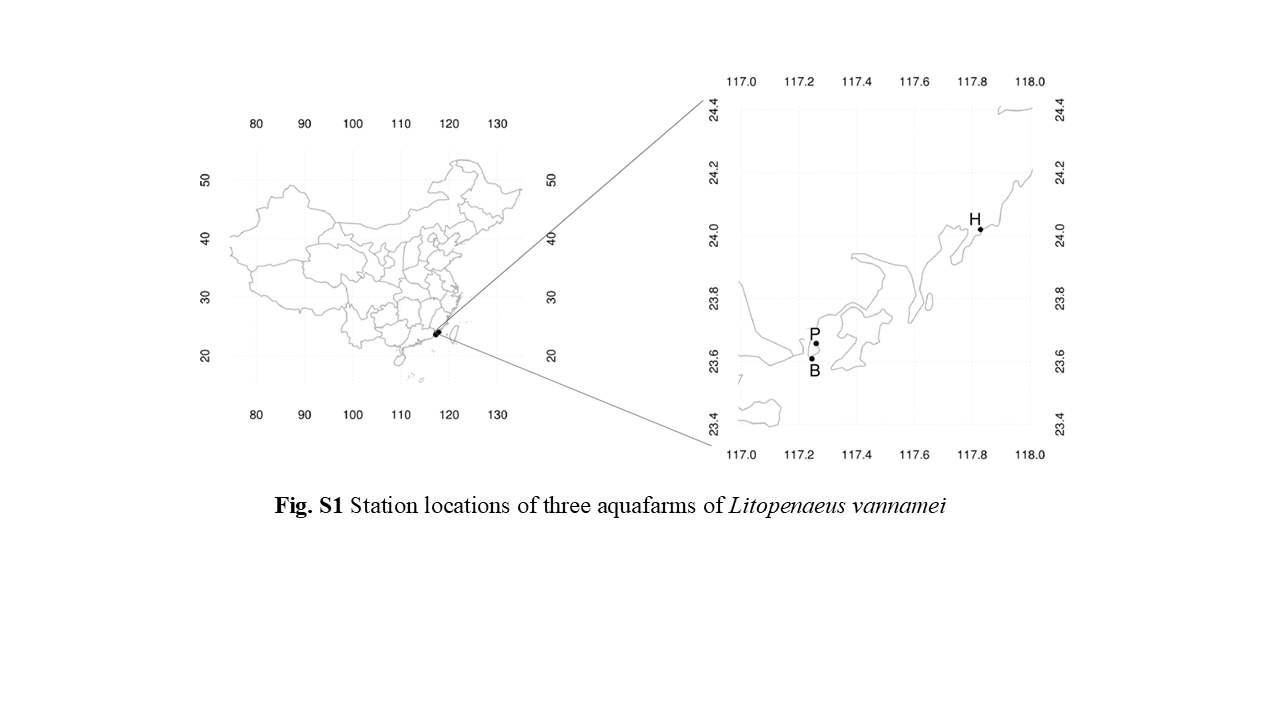

Supplement: Supplementary file 1 [file microorganisms-13-00881-s001.zip › Fig S1 .tif]
